# Metabolomic Profiling of Plasma, Urine, and Saliva of Kidney Transplantation Recipients

**DOI:** 10.3390/ijms232213938

**Published:** 2022-11-11

**Authors:** Hitoshi Iwamoto, Masaaki Okihara, Isao Akashi, Yu Kihara, Osamu Konno, Shigeyuki Kawachi, Makoto Sunamura, Masahiro Sugimoto

**Affiliations:** 1 Department of Kidney Transplantation Surgery, Hachioji Medical Center, Tokyo Medical University, Hachioji-shi, Tokyo 193-0998, Japan; 2 Department of Digestive and Transplantation surgery, Hachioji Medical Center, Tokyo Medical University, Hachioji-shi, Tokyo 193-0998, Japan; 3 Institute of Medical Science, Tokyo Medical University, Shinjuku, Tokyo 160-8402, Japan; 4 Institute for Advanced Biosciences, Keio University, Tsuruoka, Yamagata 997-0052, Japan

**Keywords:** kidney transplantation, metabolomics, biofluid, capillary electrophoresis-mass spectrometry

## Abstract

Kidney biopsy is commonly used to diagnose kidney transplant dysfunction after transplantation. Therefore, the development of minimally invasive and quantitative methods to evaluate kidney function in transplant recipients is necessary. Here, we used capillary electrophoresis-mass spectrometry to analyze the biofluids collected from transplant recipients with impaired (Group I, n = 31) and stable (Group S, n = 19) kidney function and from donors (Group D, n = 9). Metabolomics analyses identified and quantified 97 metabolites in plasma, 133 metabolites in urine, and 108 metabolites in saliva. Multivariate analyses revealed apparent differences in the metabolomic profiles of the three groups. In plasma samples, arginine biosynthesis and purine metabolism between the I and S Groups differed. In addition, considerable differences in metabolomic profiles were observed between samples collected from participants with T cell-mediated rejection (TCR), antibody-mediated rejection, and other kidney disorders (KD). The metabolomic profiles in the three types of biofluids showed different patterns between TCR and KD, wherein 3-indoxyl sulfate showed a significant increase in TCR consistently in both plasma and urine samples. These results suggest that each biofluid has different metabolite features to evaluate kidney function after transplantation and that 3-indoxyl sulfate could predict acute rejection.

## 1. Introduction

Kidney dysfunction after transplantation induces chronic rejection, which reduces long-term survival [1,2,3]. Therefore, accurate diagnosis and appropriate treatment after kidney transplantation are essential for improving patients’ prognosis and quality of life. Furthermore, patients whose kidney function did not recover to baseline after transplantation showed a worse prognosis [4]. Therefore, early diagnosis of rejection and therapeutic intervention is vital. However, an accurate diagnosis of post-transplant rejection at an early stage is difficult.

Symptoms of renal dysfunction after kidney transplantation include worsening serum creatinine, decreased urine output, fever, graft pain, and edema [5]. Imaging tests such as ultrasonography and computed tomography are employed for diagnosis. However, these tools cannot distinguish acute rejection from urinary tract infection, obstruction, drug-induced nephropathy, or acute tubular necrosis. Therefore, biopsy is used complementarily to diagnose acute rejection. However, renal allograft biopsy is not recommended in some cases; many transplant recipients take anticoagulants post-renal transplant because of cardiovascular complications, and renal transplant biopsy causes severe complications in these patients at a low rate. The risks of allograft biopsy include bleeding, damage to other organs, infection, and loss of the allograft [6,7]. Immunosuppressive drugs are administered to patients expected to have acute rejections based on blood and imaging tests; however, the diagnosis is sometimes incorrect. These patients are at a higher risk of infection due to weakened immunity. The development of minimally invasive and objective biomarkers is necessary to eliminate such cases.

Metabolomics enable the comprehensive identification and quantification of small organic molecules called metabolites. This technology has been used to analyze aberrant metabolic pathways in kidney transplantation [8,9]. To understand the systematic changes in metabolites post-renal transplantation, several spectroscopic and spectrometric-based techniques, such as nuclear magnetic resonance (NMR) [10,11,12], gas-chromatography/mass spectrometry (GC/MS) [10,13], and liquid chromatography (LC)-MS [14,15,16,17], have been used. Capillary electrophoresis-MS (CE-MS) is also a common tool to enable the simultaneous profiling of hydrophilic metabolites. This tool was used to assess renal function in chronic kidney diseases [18]; however, the applicability of CE-MS to analyze the changes in metabolites after kidney transplantation has not been explored.

Different biofluids, such as serum, urine, and saliva, are used to assess the changes in the post-renal transplantation metabolite profiles. In a previous study, the serum samples were used to monitor the transition of metabolomic patterns (i.e., metabolomic profiles) before and after kidney transplantation [12]. Kienana et al. [10] used urine samples to assess the metabolomic pattern changes in kidney transplant recipients treated with tacrolimus and cyclosporine [10]. Furthermore, new metabolite biomarkers have been explored to diagnose T-cell-mediated rejection using urine samples collected from children undergoing kidney transplantation [14]. Zhao et al. monitored the serum metabolomic profile of patients after kidney transplantation. They reported that the concentration of dehydroepiandrosterone sulfate, an adrenal androgen, was significantly lower in patients with acute rejection [15]. A similar study was conducted to analyze serum samples from patients after kidney transplantation [16]. The serum metabolomic profile was analyzed, and several metabolites, such as trimethylamine-*N*-oxide, choline, and betaine, were shown to be associated with chronic inflammation of the kidney [17]. However, the simultaneous collection of different types of biofluids and comparison of their metabolomic profiles are rare.

The purpose of this study was to conduct a metabolomic analysis of plasma, urine, and saliva samples collected from participants undergoing kidney transplantation. We employed CE-MS to monitor a wide range of hydrophilic metabolites simultaneously. We analyzed differences in the metabolomic profiles of transplant recipients with and without kidney dysfunction. The profiles of the transplant recipients with and without acute rejection were also compared.

## 2. Results

### 2.1. Comparison of Plasma Metabolomics among Donors and Kidney Transplant Recipients

The characteristics of the participants in the donor (Group D), kidney transplant recipients with stable (Group S), and impaired (Group I) kidney function groups are summarized in Table 1. Age and creatinine concentration between the three groups were significantly different (*p* = 0.024 and <0.0001, respectively). In participants who underwent kidney transplantations, the age of the donors showed a significant difference (*p* = 0.026). Metabolomics analyses identified and quantified 97 metabolites in plasma, 133 metabolites in urine, and 108 metabolites in saliva.

**Figure 1:**
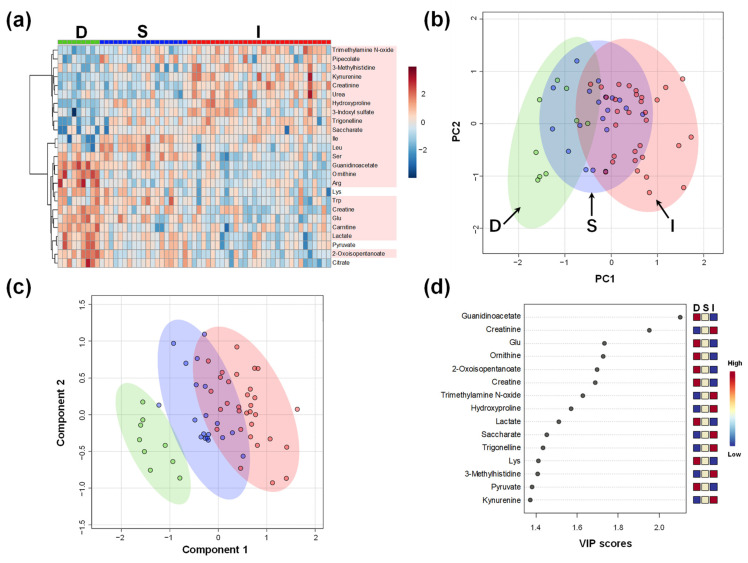
Overall metabolomic plasma profile of all participants in D, S, and I Groups. (**a**) Heatmap to visualize the metabolomic profile. Absolute concentrations are divided by the median value of each sample, log_2_-transformed, and converted to Z-score. Metabolites showing high variation among the three groups (top 25 by analysis of variance) are visualized. The metabolites showing *p* < 0.05 (ANOVA) are colored; (**b**) Score plots of principal component (PC) analysis. The contribution ratios are 20.2% and 12.7% for the first and second PC (PC1 and PC2), respectively. Green, blue, and red dots indicate data in D, S, and I Groups. The corresponding ellipses show 95% confidence intervals; (**c**) Score plots of partial least squares-discriminant analysis (PLS-DA). *R*^2^ and *Q*^2^ of leave-one-out-cross-validation are 0.82 and 0.70, respectively. The dots and ellipses are the same as the ones in the panel (**c**); (**d**) Variable importance of projection (VIP) scores of PLS-DA. D, Donor group; S, kidney transplant recipients with stable kidney function; I, kidney transplant recipients with impaired kidney function.

Figure 1 shows the overall plasma metabolomic profiles of the three groups. Clustering (Figure 1a) and principal component analysis (PCA; Figure 1b) showed the greatest distance between the metabolomic profiles of Groups D and I and that of Group S intervened among them. Partial least squares-discriminant analysis (PLS-DA) clearly discriminated between the three groups (Figure 1c). The variable importance of projection (VIP) score identified that guanidinoacetate contributed the most to the discrimination of PLS-DA, which was more prominent than that of creatinine (Figure 1d). The metabolites showing high VIP scores, e.g., guanidinoacetate and creatinine, highly contributed to the discrimination of the three groups. To validate our findings in Figure 1, we analyzed the serum creatinine concentrations independently using the enzymatic method. The creatinine concentration determined using the enzymatic method and the plasma creatinine concentration showed a clear consistency (Figure 1a).

### 2.2. Comparison of Plasma Metabolomics between Patients with Kidney Transplantations with Stable (S) and Impaired Graft (I) Kidney Functions

**Figure 2:**
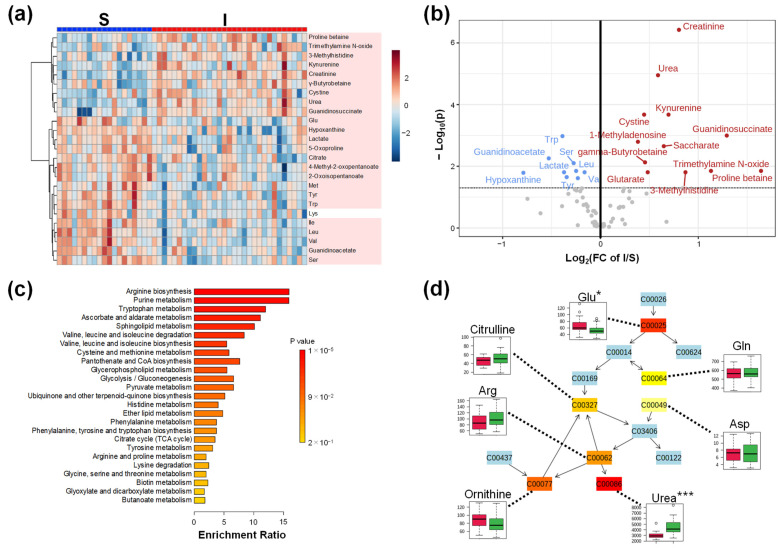
Plasma metabolomic profile of kidney transplant recipients (Groups S and I). (**a**) Heatmap to visualize the metabolomic profile. The processing is the same as the legends in Figure 1a. The metabolites showing *p* < 0.05 (Mann–Whitney test) are colored; (**b**) Volcano plots. The *x*-axis indicates the log_2_-fold change (FC) of S/I. The *y*-axis shows the −log_10_(P) of each metabolite. *p*-value was calculated using the Mann–Whitney test with false discovery rate (FDR) correction. The horizontal line indicates −log_10_(P) = 1.3, i.e., FDR-adjusted *p* = 0.05. The metabolites showing > −log_10_(P) = 1.3 are colored in red (higher in Group S) and blue (lower in Group S); (**c**) Enrichment analysis. Kyoto Encyclopedia of Genes and Genomes (KEGG) pathway was used; (**d**) Arginine biosynthesis shows the largest −log_10_(P) value by the pathway analysis. Boxplots are shown for the data-mapped metabolites. Green and red box plots show the data of Group I and S, respectively. S, kidney transplant recipients with stable kidney function; I, kidney transplant recipients with impaired kidney function. * *p* < 0.05 and *** *p* < 0.001.

Next, we compared the metabolomic profiles of Groups S and I. Clustering showed a clear difference in the metabolomic profiles between the S and I groups (Figure 2a). Except for Lys, all metabolites in the heatmap showed *p* < 0.05 (Mann–Whitney test). Volcano plots revealed that the metabolite concentrations of 12 metabolites were higher and those of 9 metabolites were significantly lower in Group I than those in Group S (Figure 2b). Creatinine showed the largest −log_10_(P), that is, the smallest *p*-value. Urea and guanidinoacetate were present in higher concentrations. Meanwhile, lactate, an end product of glycolysis, and many amino acids, such as tryptophan (Trp), serine (Ser), leucine (Leu), valine (Val), and tyrosine (Tyr), showed lower concentrations. Furthermore, the enrichment analysis showed that arginine biosynthesis had the smallest *p*-value (Figure 2c). Seven metabolites, glutamine (Gln), glutamic acid (Glu), aspartic acid (Asp), arginine (Arg), ornithine, citrulline, and urea, were mapped to this pathway (Figure 2d).

### 2.3. Comparison of Plasma, Urine, and Saliva Metabolomics of Group I

**Figure 3:**
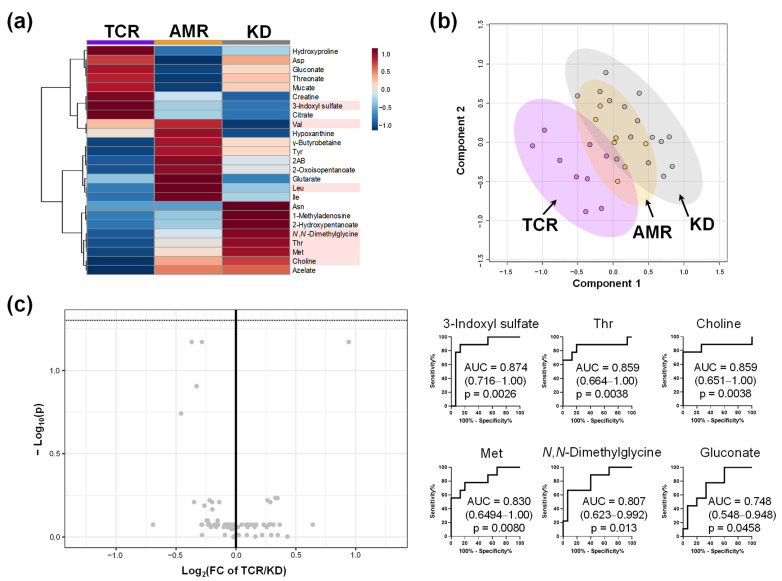
The difference in the plasma metabolomic profile of participants with kidney transplantation (groups TCR, AMR, and KD). (**a**) Heatmap of metabolomic profile. The processing is the same as the legends in Figure 1a. Subsequently, the data were averaged for each group. No metabolite shows FDR-adjusted *p* < 0.05; (**b**) Score plots of PLS-DA using the metabolites shown in panel (**a**). *R*^2^ and *Q*^2^ of leave-one-out-cross-validation are 0.82 and 0.51, respectively; (**c**) Volcano plots between TCR and KD. The horizontal line indicates −log_10_(P) = 1.3, i.e., FDR-adjusted *p* = 0.05. The area under the receiver operating characteristic (ROC) curves (AUC) is shown for the metabolites with *p* < 0.05 (without FDR correction). TCR, T cell-mediated rejection group; AMR, antibody-mediated rejection group; KD, kidney dysfunction other than rejection group.

To evaluate the specificity of the metabolomic change, the participants in Group I were pathologically classified into three groups: T cell-mediated rejection (TCR group, n = 9), antibody-mediated rejection (AMR group, n = 7), and kidney dysfunction other than rejection (KD group, n = 15) (Table 2). The characteristics of these participants are shown in Table 2. No significant differences were observed in sex, age of the patient, donor age, body mass index, or creatinine level. Clustering analysis of the plasma metabolomic profile revealed group-specific metabolite patterns (Figure 3a). For example, the concentrations of hydroxyproline, Asp, gluconate, threonate, creatine, 3-indolyl sulfate, and citrate were higher in the TCR group than those in the other groups. Although the score plots of PLS-DA showed a difference among TCR, KD, and AMR groups (Figure 3b), the volcano plot revealed no significant differences between TCR and KD groups. No metabolite showed FDR-adjusted *p* < 0.05 (Figure 3c). The receiver operating characteristic (ROC) curves of seven metabolites showing *p* < 0.05 without FDR correction were described.

**Figure 4:**
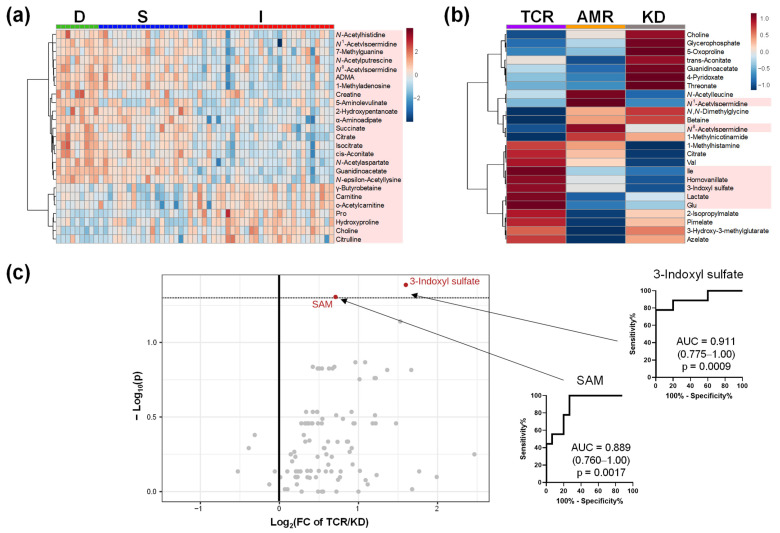
The urinary metabolomic profile. (**a**) Heatmap of groups D, S, and I; (**b**) Heatmap of groups TCR, AMR, and KD; (**c**) Volcano plots between TCR and KD. The processing is the same as the legends in Figure 1a, Figure 2b and Figure 3a. D, Donor group; S, kidney transplant recipients with stable kidney function; I, kidney transplant recipients with impaired kidney function; TCR, T cell-mediated rejection group; AMR, antibody-mediated rejection group; KD, kidney dysfunction other than rejection group.

**Figure 5:**
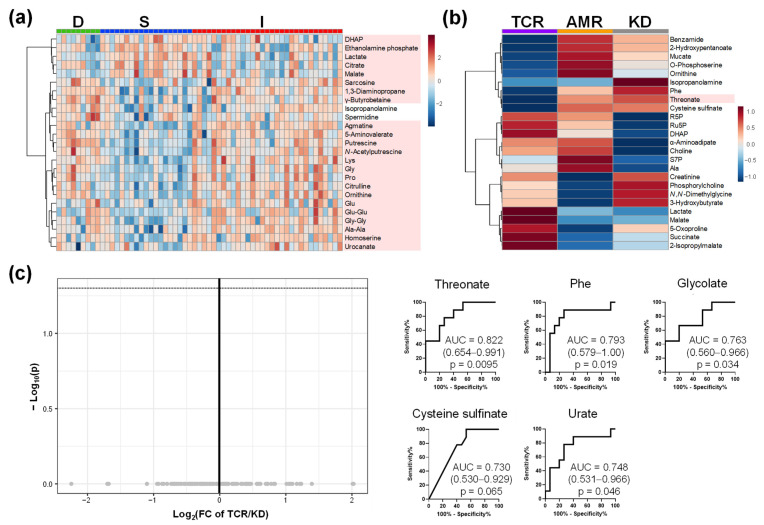
The salivary metabolomic profile. (**a**) Heatmap of groups D, S, and I. (**b**) Heatmap of groups TCR, AMR, and KD. (**c**) Volcano plots between TCR and KD. The processing is the same as the legends in Figure 1a, Figure 2b, and Figure 3a. D, Donor group; S, kidney transplant recipients with stable kidney function; I, kidney transplant recipients with impaired kidney function; TCR, T cell-mediated rejection group; AMR, antibody-mediated rejection group; KD, kidney dysfunction other than rejection group.

The metabolomic profiles of urine and saliva are shown in Figure 4 and Figure 5, respectively. The differences among groups D, S, and I are shown (Figure 4a and Figure 5a). The metabolomic profiles of patients in the TCR, AMR, and KD groups are shown in Figure 4b and Figure 5b. Volcano plots and ROC curves between TCR and KD are shown in Figure 4c and Figure 5c. The concentrations of two urinary metabolites, 3-indoxyl sulfate and S-adenosyl methionine (SAM) were significantly higher in TCR group than those in KD group (FDR-adjusted *p* < 0.05). However, the concentrations of none of the salivary metabolites differ significantly between the TCR and KD groups. Among the three biofluids, urine samples identified the largest number of significantly different metabolites between the TCR and KD groups.

### 2.4. Comparison of Metabolome Profiles in Patients with Kidney Transplantation Grouped by Banff Classification

The Banff Classification of Allograft Pathology is a standardized working classification system for a universal grading system to assess graft injuries. In this study, we evaluated the following Banff classification factors to characterize the participants with kidney transplantation: (1) interstitial (i) and tubulitis (t) for TCR, (2) C4 complement component (c4d) for AMR [19], and (3) aah for calcineurin inhibitor (CNI)-induced nephrotoxicity [20]. Positive cases were defined as i + t ≥ 2, c4d ≥ 1, and aah ≥ 1, and the participants were divided into positive (+) and negative (−) subgroups.

#### 2.4.1. Comparison of (i + t)

**Figure A2:**
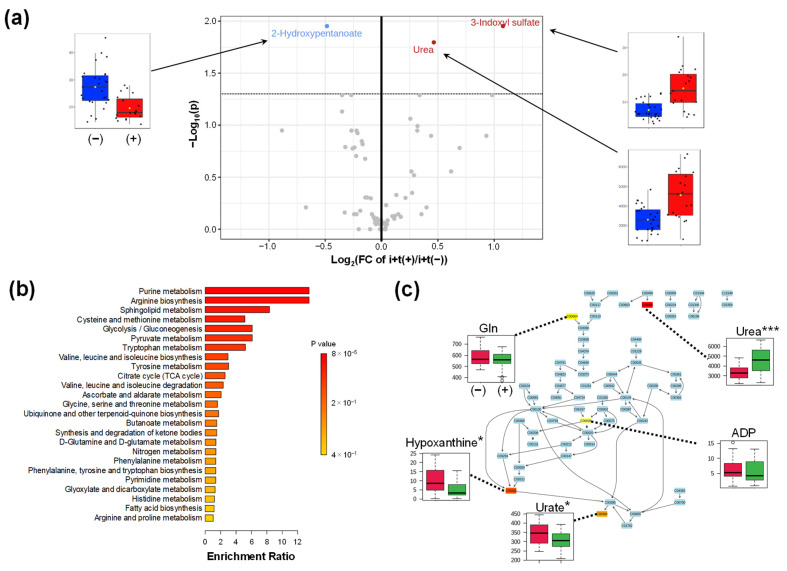
The difference in the metabolomic profile of participants with kidney transplantation (groups i + t (+) and i + t (−)). (**a**) Volcano plots. The *x*-axis indicates the log_2_-fold change (FC) of i + t (+)/i + t (−). The *y*-axis shows the −log_10_(P) of each metabolite. False discovery rate (FDR)-adjusted *p*-value was calculated using the Mann–Whitney test. Metabolites showing *p* < 0.05 with FDR correction are shown using box plots. Red and blue box plots show the data of groups i + t (+) and i + t (−), respectively; (**b**) Enrichment analysis; (**c**) Purine metabolism showing the largest −log_10_(P) value by the pathway analysis. Green and red box plots show the data of groups i + t (+) and i + t (−), respectively. * *p* < 0.05 and *** *p* < 0.001.

The characteristics of patients with kidney transplantation in the i + t (+) and i + t (−) subgroups are shown in Table A1. The comparative metabolomic profiles are shown in Figure A2. Volcano plots show 3-indoxyl sulfate and urea with significantly higher concentrations and 2-hydroxypentanoate with substantially lower concentrations in the i + t (+) group (Figure A2a). Enrichment analysis identified the purine metabolism pathway with the smallest *p*-value (Figure A2b). The quantified data of Gln, urea, urate, hypoxanthine, and ADP were mapped in this pathway (Figure A2c).

#### 2.4.2. Comparison of c4d

**Figure A3:**
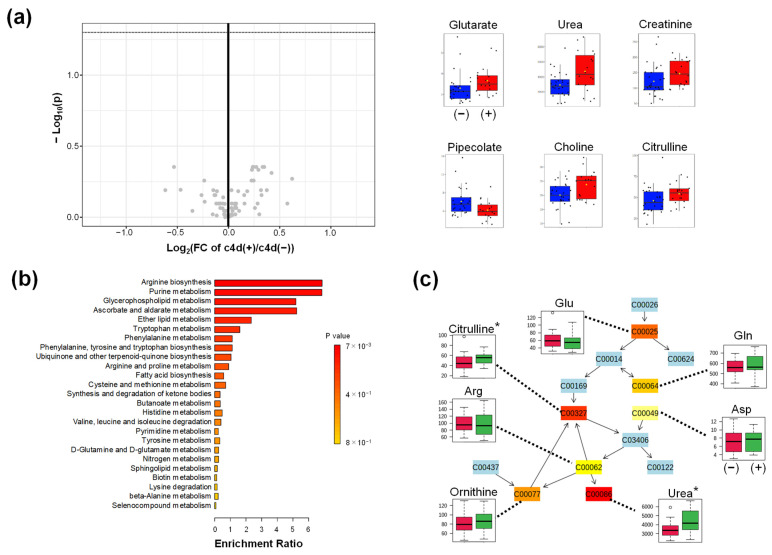
The difference in the metabolomic profile of participants with kidney transplantation (groups c4d (+) and c4d (−)). (**a**) Volcano plots. The *x*-axis indicates the log_2_-fold change (FC) of c4d (+)/c4d (−). The *y*-axis shows the −log_10_(P) of each metabolite. FDR-adjusted *p*-value was calculated using the Mann–Whitney test. Metabolites showing *p* < 0.05 without correction are shown using box plots. Red and blue box plots show the data of c4d (+) and c4d (−), respectively; (**b**) Enrichment analysis; (**c**) Arginine biosynthesis showing the largest −log_10_(P) value by the pathway analysis. Green and red box plots show the data of c4d (+) and c4d (−), respectively. * *p* < 0.05.

Table A2 represents the characteristics of the patients with kidney transplantation in the c4d (+) and c4d (−) subgroups. Volcano plots of the metabolites in c4d (+) and c4d (−) subgroups showed no different significant metabolites with FDR-adjusted *p* < 0.05. Five metabolites with higher concentrations and one metabolite with lower concentrations were identified in the c4d (+) group with *p* < 0.05 without FDR correction (Figure A3a). Enrichment analysis identified purine metabolism with the smallest *p*-value (Figure A3b,c).

#### 2.4.3. Comparison of aah

**Figure A4:**
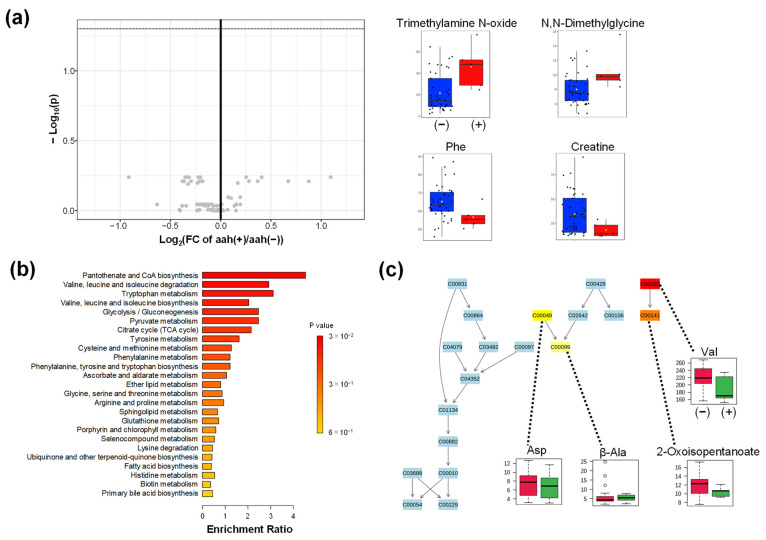
The difference in the metabolomic profile of participants with kidney transplantation (groups S aah (+) and aah (−)). (**a**) Volcano plots. The *x*-axis indicates the log_2_-fold change (FC) of aah (+)/aah (−). The *y*-axis shows the −log_10_(P) of each metabolite. FDR-adjusted *p*-value was calculated using the Mann–Whitney test. Metabolites showing *p* < 0.05 without correction are shown using box plots; (**b**) Enrichment analysis; (**c**) Pantothenate and CoA biosynthesis showing the largest −log_10_(P) value by the pathway analysis.

The characteristics of the patients in the aah (+) and aah (−) subgroups did not differ significantly (Table A3). The comparative metabolomic profiles of the aah (+) and aah (−) subgroups are shown in Figure A4. Volcano plots showed no significant difference in the concentration of the identified metabolites with FDR-adjusted *p* < 0.05. However, two metabolites with higher concentrations and two metabolites with lower concentration were identified in the aah (+) subgroup with *p* < 0.05 without FDR correction (Figure A4a). Enrichment analysis identified pantothenate and CoA biosynthesis had the smallest *p*-value (Figure A4b). Val, Asp, β-alanine (β-Ala), and 2-oxisopenntaonate were identified in this pathway (Figure A4c).

## 3. Discussion

With advancements in the techniques, patient and graft survival rates of kidney transplants have improved. However, the prognosis for the long-term is relatively poor compared to the short-term [21]. For instance, short-term prognoses are excellent for the Japanese data. i.e., one-year and five-years graft survival rates for living donors were 98.7% and 94.3%, respectively. Conversely, 10-year and 15-year graft survival rates were 85.2% and 73.9%, respectively. The grafts are affected by ischemia-reperfusion injury, rejection, recurrence of the primary disease, immunosuppressive drugs, etc., and transplanted kidneys develop chronic allograft injury due to these factors. Chronic allograft injury may result from recurrent and de novo glomerulonephritis, BK polyomavirus-associated nephropathy, chronic active antibody-mediated rejection, chronic active T cell-mediated rejection, or renal artery rejection stenosis [22,23]. Even without rejection, the graft is vulnerable to chronic insults, such as dose-dependent nephrotoxicity associated with long-term CNI therapy, which may lead to deteriorating kidney function. Managing CNI toxicity is the cornerstone for improving the long-term graft survival rate [24]. For this purpose, a diagnostic biopsy is commonly used. However, a more minimally invasive diagnosis is preferable; therefore, metabolomic analysis of the biofluid samples was conducted. Since rejection leads to deterioration of the long-term prognosis of transplantation, an early and accurate diagnosis of rejection is preferable [23]. Therefore, we explored the identification of biomarkers to discriminate between TCR and KD.

The comparison of plasma metabolomic profiles between donors (Group D) and participants with stable (Group S) and impaired (Group I) kidney function after kidney transplantation showed apparent differences. The differences in the metabolomic profiles between Groups S and I included a significantly higher creatinine concentration in Group I than that in Group S. In addition, urea, glutamate, and trimethylamine *N*-oxide levels were substantially higher in Group I. In contrast, amino acids (Trp, Ser, Leu, Val, and Thr) and lactate levels were significantly lower in Group I than those in Group S.

An NMR-based metabolite profiling study to evaluate kidney function using eGFR revealed that lower glutamine, Phe, Thr, His, and Pro levels decreased kidney function in the study participants [25]. Concordantly, our study also showed a consistent change in Thr, and the levels of most amino acids were higher in Group I than that in Group S. The kidney has a unique metabolomic function that transforms phenylalanine to tyrosine by hydroxylation and provides Thr to the whole body [26,27]. Taken together, the decrease in plasma Thr level is one of the phenotypes attributed to decreased kidney function.

Our findings demonstrated that creatinine and urea were the first and second significantly higher abundant metabolites in Group S than in Group I. According to pathway analysis, these metabolites belong to the arginine biosynthesis pathway, which was also identified as the most differently enriched pathway between S and I. In this pathway, glutamate was significantly decreased, whereas the other amino acids, including Arg, Asp, and Gln, showed no significant differences. Arg is a precursor of the key metabolites, asymmetric dimethylarginine (ADMA) and symmetric dimethylarginine (SDMA), which play critical roles in endothelial dysfunction. Arg residues in proteins can be singly or doubly methylated post-translationally, and the proteolysis of arginine-methylated proteins results in monomethyl arginine. ADMA and SDMA are risk indicators for cardiovascular disease and death in various pathologies, including kidney disease [28]. In addition, these metabolites have been used to evaluate kidney function in kidney transplantation [29,30]. However, in our plasma metabolomic data, the concentrations of these metabolites did not differ significantly between Groups S and I.

TCR, AMR, and KD comparisons also showed specific profiles. Metabolites showing significant differences between the TCR and KD were considered predictors of acute rejection. In total, six metabolites showed different concentrations in the plasma (*p* < 0.05 without FDR correction); the concentrations of 3-indoxyl sulfate and gluconate were significantly increased, and those of *N*,*N*-dimethylglycine, choline, Thr, and Met were significantly decreased in the TCR group compared to the KD group. However, these differences were FDR-adjusted *p*-values > 0.05. In urine samples, the concentrations of two metabolites, including 3-indoxyl sulfate and SAM, were significantly higher in TCR group than those in KD group (FDR-adjusted *p*-values < 0.05). In saliva samples, five metabolites showed significantly lower concentrations in TCR than in KD, while all differences showed were FDR-adjusted *p*-values > 0.05. Plasma and urine samples showed different metabolite patterns; however, only 3-indoxyl sulfate was consistently increased in both samples. This metabolite is a tubular toxin that induces apoptotic and necrotic death of tubular cells in the kidney [31]. It stimulates glomerular sclerosis [32] and interstitial fibrosis [31], which induce kidney failure. In our data, 3-indoxyl sulfate showed the highest discrimination ability to discriminate TCR from KD in plasma (AUC = 0.874; 0.716–1.00; *p* = 0.0026) and in urine (AUC = 0.911; 0.755–1.00; *p* = 0.0009), which showed potential as a biomarker of this metabolite to predict acute rejection. However, the metabolites showing differences but FDR-adjusted *p* > 0.05 levels should be validated their discrimination abilities using a larger cohort.

We also analyzed the relationship between plasma metabolomic profiles and Banff classification, which is the basis for the diagnosis of kidney dysfunction. The subgroup i + t (+) showed higher concentrations of 3-indoxyl sulfate in urine than in the subgroup i + t (−), similar to the comparison between TCR and KD. Compared to the c4d (−) subgroup, the c4d (+) subgroup had higher concentrations of urea, creatinine, and choline. The concentrations of trimethylamine *N*-oxide and *N*,*N*-dimethylglycine were higher in the aah (+) subgroup than those in the aah (−) subgroup. Trimethylamine *N*-oxide is mainly formed from various nutritional metabolites, including choline, creatinine, and dimethylglycine [33], and is known to be associated with kidney function [17]. Thus, the i + t classification mainly reflected the 3-indoxyl sulfate-related pathways, and c4d and aah classifications reflected the aberrance of trimethylamine *N*-oxide-related pathways derived from kidney functions; however, there were overlaps, for example, higher citrulline was observed in both i + t and c4d classifications.

This study has several limitations. This study included small sample size, and validation of the other cohort is necessary to confirm the generalization ability; in particular, AUC values should be rigorously validated to assess the discrimination ability of the identified metabolites as biomarkers. The sample collection time was dependent on surgery. Diurnal variation in the metabolomic profiles of each subject was not eliminated. The age of Group D was significantly higher than that of the other groups. In the transplantation recipients, donor age also showed significant differences. Thus, the current analysis does not eliminate the effects of these cofactors. The serum creatinine concentrations measured by the enzymatic method and the plasma creatinine concentration showed a clear consistency. However, the validations of urinary and salivary metabolomic profiles with the data with other methods are necessary to confirm the reliability of the quantified data in this study.

## 4. Material and Methods

### 4.1. Study Design

We retrospectively included kidney transplant recipients from the Department of Kidney Transplantation at Hachioji Medical Center, Tokyo Medical University (Hachioji, Japan). The study protocol was approved by the Ethics Committee of the Tokyo Medical University Hachioji Medical Center (no. 2478). Patients with kidney transplant followed up at our hospital were divided into three groups as follows: kidney transplant donors (Group D, n = 9), kidney transplant recipients with stable kidney function after kidney transplantation (Group S, n = 19), and those with impaired graft kidney function after transplantation (Group I, n = 31).

A definitive diagnosis was made using a kidney graft biopsy. All biopsies were performed using a 16-gauge spring-loaded biopsy needle under ultrasound guidance. The same pathologist scored the biopsies according to the Banff ‘09 classification, blinded to the biomarker metabolites [34]. Blood, urine, and saliva samples from Group D were collected immediately before surgery. Unstimulated whole saliva was collected and immediately stored at −80 °C. Blood samples were collected with ethylenediaminetetraacetic acid-2Na (EDTA-2Na) as an anticoagulant and stored at 4 °C immediately after collection. The samples were centrifuged for 15 min (1500*× g* at 4 °C), divided into aliquots, and preserved at −80 °C. Urine samples were immediately stored at −80 °C. Tissue samples from Group D were collected at 0 h biopsies. The samples in Group S were collected at the protocol biopsy, performed three months post-transplant. The samples in Group I were collected during episode biopsy when renal function began to deteriorate. Maintenance immunosuppression consisted of a CNI (tacrolimus or cyclosporine), mycophenolate, and corticosteroids with initial antibody induction using basiliximab. eGFR was calculated using the modified IDMS-MDRD study equation [35].

### 4.2. Sample Processing for Metabolomic Analysis

Metabolomic analyses of plasma samples were conducted as previously reported [36] with slight modifications. Briefly, frozen plasma samples were thawed. To 40 μL plasma samples, 360 μL methanol and 20 μM each of methionine sulfone, 2-(*N*-morpholino)ethanesulfonic acid, and D-camphor-10-sulfonic acid were added, and the preparation was mixed well. Next, 160 μL deionized water and 400 μL chloroform were added, and the solution was centrifuged at 10,000× *g* for 3 min at 4 °C. The upper aqueous layer was filtered through a Millipore 5-kDa cut-off filter at 9100× *g* for 180 min at 4 °C to remove large molecules. The remaining solution was then concentrated by centrifugation (960× *g*) for 3 h at 40 °C, and samples were lyophilized until required for capillary electrophoresis time-of-flight mass spectrometry (CE-TOFMS) analyses. For metabolite analysis, samples were dissolved in 40 μL Milli-Q water containing 200 μM each of 3-aminopyrrolidine and trimesic acid for CE-TOF-MS. Saliva and urine samples were processed following the procedures described in previous studies [37,38,39].

### 4.3. Instrument of Metabolomic Analysis

All CE-TOF-MS experiments were performed using an Agilent CE capillary electrophoresis system (Agilent Technologies, Waldbronn, Germany), Agilent G1969A and G6220A Accurate-Mass TOF LC-MS system (Agilent Technologies, Palo Alto, CA, USA), Agilent 1100 and 1200 series isocratic high-performance LC pumps, G1603A Agilent CE-MS adapter, and Agilent CE electrospray ionization (ESI)-MS sprayer kit (G1600AX and G7100A). An Agilent G1607-60001 platinum ESI needle was used for anion analysis. The Agilent ChemStation software (ver. A.10.02, B.02.01.SR1, and B.03.02, C.01.07.SE1, Agilent Technologies, Waldbronn, Germany) for CE and the Agilent MassHunter software (ver. B.02.00, Agilent Technologies, Palo Alto, CA, USA) was used for the system control and data acquisition.

### 4.4. CE-TOFMS Conditions for Cationic Metabolite Analysis

Separations were performed in a fused silica capillary (50 mm i.d. ×96 cm and 50 mm i.d.  ×97 cm total length; Sakata Rika, Yamagata, Japan). For preconditioning, the capillary was filled with 1 M formic acid (run buffer) as the electrolyte for 4 min for plasma and urine analysis. The capillary was filled with ammonium formate for 5 min, Milli-Q for 5 min, and run buffer for 5 min for saliva analysis. Approximately 5 nL of the sample solution was injected at 50 mbar for 5 s, and a voltage of 30 kV was applied. The capillary temperature was maintained at 20 °C, and the sample tray was cooled to below 4 °C. Methanol–water (50% *v*/*v*) containing 0.1 μM hexakis(2,2-difluoroethoxy)phosphazene was used as the sheath liquid at 10 μL/min. ESI-TOFMS was performed in the positive ion mode, and the capillary voltage was set at 4000 V. The flow rate of the heated dry nitrogen gas (heater temperature: 300 °C) was maintained at 7 psig. In the TOFMS, the fragmentor, skimmer, and octapole radio frequency voltages (Oct RFV) were set at 75, 50, and 125 V, respectively. The automatic recalibration of each acquired spectrum was performed using the masses of the reference standards [13C isotopic ion of a protonated methanol dimer (2MeOH + H)]+, *m*/*z* 66.063061 and [hexakis(2,2-difluoroethoxy)phosphazene + H]^+^, *m*/*z* 622.028963. The exact mass data were acquired at 1.5 spectra/s over the *m/z* range of 50–1000.

### 4.5. CE-TOFMS Conditions for Anionic Metabolite Analysis

A commercially available COSMO (+) capillary (50 mm i.d. × 105 cm; Nacalai Tesque, Kyoto, Japan) chemically coated with a cationic polymer was used as the separation capillary. For preconditioning, the capillary was filled with 50 mM ammonium acetate (pH 3.4 for 2 min, and the buffer was run for 5 min. A 50 mM ammonium acetate solution (pH 8.5) was used as the electrolyte solution for CE separation. The sample solution (30 nL) was injected at 50 mbar for 30 s, and a voltage of 30 kV was applied. Ammonium acetate (5 mM) in 50% methanol–water (*v*/*v*) containing 0.1 μM hexakis was delivered as the sheath liquid at 10 μL/min. ESI-TOFMS was conducted in negative ion mode; the capillary voltage was set at 3500 V. For TOFMS, the fragmentor, skimmer, and Oct RFV were set at 100, 50, and 200 V, respectively. Automatic recalibration of each acquired spectrum was performed using the masses of reference standards ([13C isotopic ion of deprotonated acetic acid dimer (2CH_3_COOH-H)]^−^, *m*/*z* 120.038339), and ([hexakis + deprotonated acetic acid (CH_3_COOH-H)]^−^, *m*/*z* 680.035541). Exact mass data were acquired at 1.5/s over an *m*/*z* range of 50–1000. The other conditions were identical to those used in cationic metabolite analyses.

### 4.6. Data Analysis

The raw data were processed using MasterHands (ver. 2.17.1.11, Keio University, Yamagata, Japan) [40]. A concentration matrix with the identified metabolite names was used for subsequent statistical analyses. Absolute concentrations were used for plasma and saliva samples. The concentration divided by the creatinine concentration quantified in the metabolomic analyses was used for the urine samples. Hierarchical clustering and PCA were performed to visualize the overall metabolomic profiles. PLS-DA was used as a supervised discrimination method. Discriminant accuracy was assessed by the *R*^2^ value, and generalization ability was evaluated by *Q*^2^ values. Kruskal–Wallis and Mann–Whitney tests were used to compare quantitative values for two- and three-group comparisons. *p*-values were corrected by the false discovery rate (Benjamini–Hochberg) method to consider the multiple independent tests. The AUC was used to evaluate the discriminating ability of metabolites between the two groups.

The MetaboAnalyst (ver. 5.0, https://www.metaboanalyst.ca/ (accessed on 1 June 2022)) [41] and JMP Pro (ver. 16.0.0, Cary, NC, USA) were used for these analyses. A detailed description of the metabolomics data analysis using MetaboAnalyst is described in Appendix B.

## 5. Conclusions

We analyzed the metabolomic profiles of saliva, plasma, and urine collected from kidney transplant recipients and donors using CE-MS. Among the recipients, impaired and stable kidney functions showed clear differences, including creatinine, urea, and various metabolites in plasma samples. TCR, AMR, and KD also showed clear differences in metabolomic profiles. Plasma, urine, and saliva showed different metabolomic patterns, whereas the concentration of 3-indoxyl sulfate consistently increased in the plasma and urine samples. This metabolite could be used as a potential biomarker for acute rejection. A comparison of each Banff classification showed the aberrance of 3-indoxyl sulfate and trimethylamine *N*-oxide-related pathways, indicating the reflection of metabolic changes associated with kidney function. These metabolites also have the potential to be used to evaluate acute rejection.

## Figures and Tables

**Table 1:** Characteristics of all participants in the three groups.

Item	D	S	I	*p*-Value
n	9	19	31	
Sex (Male/Female)	3/6	11/8	22/9	0.12 ^a^
Age	64 (43–67)	49 (23–74)	47 (30–68)	0.024 *^b^
BMI	23.9 (±3.73)	22.2 (±3.49)	23.2 (±3.18)	0.40 ^b^
Creatinine, mg/dL	0.68 (±0.15)	1.1 (±0.35)	2.1 (±0.71)	<0.0001 ***^b^
eGFR, mL/min/1.73 m^2^	82.2 (±18.7)	57.2 (±12.2)	28.8 (±9.01)	
Donor’s age		47 (20–68)	59 (5–72)	0.026 *^c^
Living-donor (%)		18 (94.7)	30 (96.8)	0.72 ^a^
DM (%)		6 (31.6)	12 (38.7)	0.46 ^a^
ABO-I (%)		4 (21.1)	12 (38.7)	0.13 ^a^
HD duration, months		16 (1–262)	5.5 (1–250)	0.17 ^c^
HLA A, B, DR mismatch		2 (±1.6)	3.2 (±1.4)	0.0092 **^c^
HD (%)		10 (52.6)	20 (64.5)	0.55 ^a^
PEKT (%)		8 (42.1)	9 (29.0)	0.30 ^a^
Tac (%)		11 (57.9)	15 (48.4)	0.51 ^a^
MMF (%)		19 (100)	30 (96.8)	0.43 ^a^

Mean (±standard deviation; SD) or median (min-max) are presented. BMI, body mass index; DM, diabetes mellitus; ABO-I, ABO-incompatible donor; HD, hemodialysis; PEKT, pre-emptive kidney transplantation; Tac, tacrolimus; MMF, mycophenolate mofetil; D, Donor group; S, kidney transplant recipients with stable kidney function; I, kidney transplant recipients with impaired kidney function; ^a^ χ^2^ test; ^b^ Kruskal–Wallis test; and ^c^ Mann–Whitney test, * *p* < 0.05; ** *p* < 0.01; *** *p* < 0.01.

**Table 2:** Characteristics of participants who underwent kidney transplantation.

Item	TCR	AMR	KD	*p*-Value
n	9	7	15	
Sex (Male/Female)	6/3	4/3	12/3	0.52 ^a^
Age	47 (34–65)	60 (42–66)	47 (30–68)	0.48 ^b^
Donor’s age	54 (5–72)	55 (36–66)	61 (38–71)	0.32 ^b^
BMI	23.4 (±3.71)	24.0 (±4.16)	23.7 (±2.41)	0.69 ^b^
Banff score				
i + t	4.3 (±0.5)	1.7 (±0.8)	1.5 (±1.2)	
c4d	0.7 (±1.0)	1.3 (±1.1)	0.9 (±1.2)	
aah	0 (±0)	0.3 (±0.8)	0.5 (±0.8)	

^a^ χ^2^ test; ^b^ Kruskal–Wallis test T cell-mediated rejection (TCR), antibody-mediated rejection (AMR), kidney dysfunctions other than rejection (KD), interstitial (i), tubulitis (t), and C4 complement component (c4d).

**Figure A1:**
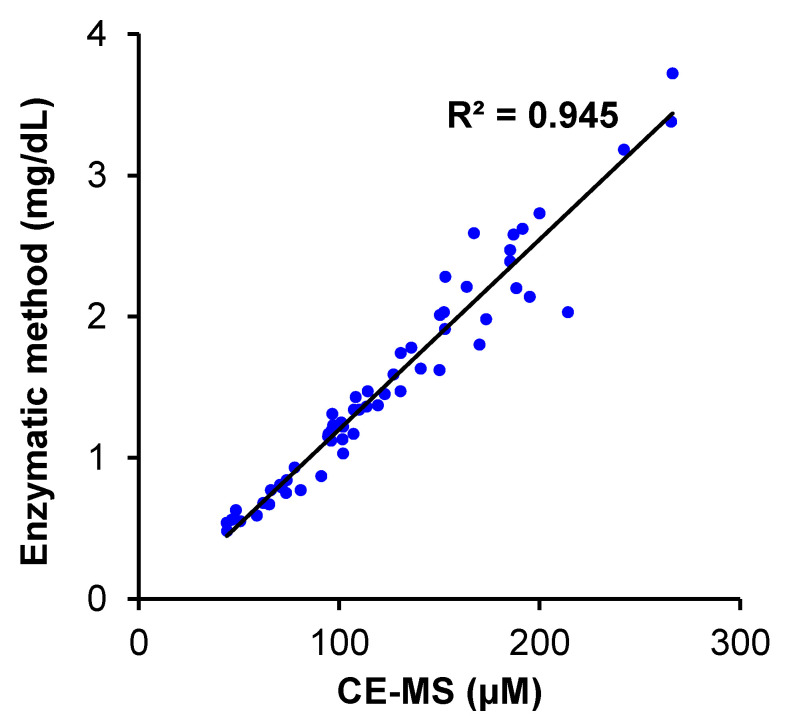
Comparison of creatinine concentration of enzymatic method and metabolomic profile. The correlation (Pearson correlation) shows *p* < 0.0001.

## Data Availability

The data presented in this study are available upon request from the corresponding author.

## Appendix A

**Table A1:** Characteristics of participants with i + t (+) and i + t (−).

Item	i + t (−)	i + t (+)	*p*-Value
N	27	19	
Sex, Male/Female	20/7	10/9	0.13 ^a^
Age	48 (23–74)	54 (34–74)	0.14 ^b^
Donor’s age	53 (20–71)	54 (5–72)	0.98 ^b^
BMI	22.4 ± 3.47	23.4 ± 3.31	0.65 ^b^
Creatinine	1.53 ± 0.72	1.73 ± 0.58	0.20 ^b^

^a^ χ^2^ test; ^b^ Mann–Whitney test.

**Table A2:** Characteristics of participants with c4d (+) and c4d (−).

Item	c4d (−)	c4d (+)	*p*-Value
n	28	18	
Sex, Male/Female	18/10	12/6	0.87 ^a^
Age	49.5 (26–74)	51 (23–68)	0.83 ^b^
Donor’s age	48 (5–72)	55.5 (26–67)	0.44 ^b^
BMI	22.7 ± 3.62	22.9 ± 3.13	0.97 ^b^
Creatinine	1.50 ± 0.74	1.75 ± 0.51	0.027 *^b^

^a^ χ^2^ test; ^b^ Mann–Whitney test. * *p* < 0.05.

**Table A3:** Characteristics of participants with aah (+) and aah (–).

Item	aah (−)	aah (+)	*p*-Value
n	41	5	
Sex, Male/Female	25/5	16/0	0.084 ^a^
Age	50 (23–74)	42 (30–68)	0.78 ^b^
Donor’s age	53 (5–72)	54 (36–71)	0.76 ^b^
BMI	22.9 (±3.45)	22.1 (±3.21)	0.75 ^b^
Creatinine	1.54 ± 0.64	2.1 ± 0.71	0.13 ^b^

^a^ χ^2^ test; ^b^ Mann–Whitney test.

## Appendix B

The metabolomics data analysis using MetaboAnalyst was performed following the procedures described below.

1. The MetaboAnalyst database was accessed from https://www.metaboanalyst.ca/ (accessed on 1 June 2022) and started by pressing “Click here to start” and selecting “Statistical Analysis [one factor]”;
2. The data matrix of quantified concentrations (metabolite × sample) was uploaded. (The first line and the second line include sample and group names. The first line includes metabolite names. The other cells include the quantified values);
3. The “None” option was selected for normalization, data transformation, and data scaling options. Then, “Normalize” and “Proceed” to go to the statistics page were clicked;
4. Afterward, the “heatmap” menu was clicked, and the following options were selected: Data source: Normalized data, Standardization: Autoscale features, Distance measure: Euclidean; clustering methods: Ward; Color contrast: Default. The “use top 25” option with the T-test/ANOVA method was checked, and the option to “Submit” was clicked to show the heatmap;
5. Then, the “volcano plot” menu was clicked. The following options—Analysis: Unpaired. Non-parametric test: Yes, *p*-value threshold: 0.05 with FDR, Group variance: Equal, were selected. Then “Submit” was pressed to show a volcano plot.
6. Then, the “PLS-DA” menu was clicked. The “Cross-Validation” tab was opened. We selected the following options: Maximum components to search: 5, Cross-validation method: 10-fold CV: Performance measure: *Q*^2^. Then, “Update” to measure *R*^2^ and *Q*^2^ was clicked. Score plots and VIP scores were obtained using the “2D Scores Plots” and “Imp. Features” tab;
7. Then, “Home” menu and “Click here to start” were clicked, and “Enrichment analysis” was selected;
8. Afterward, we opened the “Quantitative Enrichment Analysis” tab, uploaded the data matrix, and performed the data processing as described in step 3;
9. Finally, the “KEGG” option was selected in the “pathway-based analysis” menu, and the “Submit” option was clicked to conduct an enrichment analysis.
